# Vibrio cholerae serogroup O5 was responsible for the outbreak of gastroenteritis in Czechoslovakia in 1965

**DOI:** 10.1099/mgen.0.001282

**Published:** 2024-09-05

**Authors:** Caroline Rouard, Elisabeth Njamkepo, Marie-Laure Quilici, Scott Nguyen, Victoria Knight-Connoni, Renáta Šafránková, Francois-Xavier Weill

**Affiliations:** 1 Institut Pasteur, Université Paris Cité, Unité des Bactéries pathogènes entériques, Centre National de Référence Vibrions et Choléra, Paris, France; 2 ATCC, 10801 University Boulevard, Manassas, VA 20110, USA; 3 Czech National Collection of Type Cultures, National Institute of Public Health, Prague, Czech Republic

**Keywords:** 1965, Czechoslovakia, gastroenteritis, O5, *Vibrio cholerae*

## Abstract

Several authors have attributed the explosive outbreak of gastroenteritis that occurred in Czechoslovakia in 1965 to a toxigenic strain of *Vibrio cholerae* serogroup O37 based on unverified metadata associated with three particular strains from the American Type Culture Collection. Here, by sequencing the original strain preserved at the Czech National Collection of Type Cultures since 1966, we show that the strain responsible for this outbreak was actually a *V. cholerae* O5 that lacks the genes encoding the cholera toxin, the toxin-coregulated pilus protein and *Vibrio* pathogenicity islands present in *V. cholerae* O37 strains.

## Data Summary

The authors confirm that all the supporting data are provided within the article or in the supplementary data files. The short-read sequence data generated in this study have been submitted to the European Nucleotide Archive (https://www.ebi.ac.uk/ena/) under accession number ERR13133351. The complete genome sequences obtained have been deposited in GenBank (https://www.ncbi.nlm.nih.gov/genbank/) under accession numbers CP147726 and CP147727.

## Introduction

*Vibrio cholerae* is a Gram-negative bacterium with more than 200 described serogroups [1,2]. Strains of serogroups O1, and more rarely O139, are associated with cholera when they produce the cholera toxin [3]. Strains of other serogroups within this species can also be associated with clustered or sporadic cases of gastroenteritis. This latter situation is illustrated by the foodborne outbreak of gastroenteritis caused by a non-agglutinable (NAG) or non-cholera *Vibrio* (NCV) *V. cholerae* strain described by Eva Aldová and coworkers in 1968 [4]. This outbreak affected at least 56 patients (mostly young men) at a training centre for automobile industry workers in Nitra, Czechoslovakia (now Slovakia), in the autumn of 1965. After sharing a meal including French potato gratin – a traditional Czech recipe – prepared at least 10 h before the meal and served without reheating, the patients suffered gastrointestinal symptoms (mostly diarrhoea but without blood or mucus in the stools) within 20 to 30 h of the meal. The patients were treated with sulfonamides, and the symptoms had resolved by the second day in most cases. However, six severe cases were hospitalized, with no deaths or sequelae. At the time, *V. cholerae* cultures were routinely distinguished via agglutination tests using sera prepared against only the Ogawa and Inaba serotypes of *V. cholerae* (now classified as *V. cholerae* O1, classical biotype), with further classification on the basis of their metabolic pathways. The representative strain of this outbreak, NCV 10125, was described as a Heiberg group II *Vibrio* (metabolism of sucrose but not of mannose or arabinose) and was deposited in the Czech National Collection of Type Cultures (CNCTC) by Aldová in 1966 under the name CNCTC 6536. Two subsequent publications stated that the 1965 Czechoslovakian outbreak was caused by a *V. cholerae* strain from serogroup O5 without providing data to support this claim [5,6].

Curiously, between 1998 and 2023, several other studies mentioned that *V. cholerae* strain 280 NAG (American Type Culture Collection (ATCC) 25872), which is deposited at ATCC, was a representative strain from the 1965 Czechoslovakian outbreak [7,10] belonging to serogroup O37 [8,10]. According to the ATCC website (https://www.atcc.org/products/25872), this strain was deposited at ATCC by Oscar Felsenfeld, who had initially received it from Aldová (original name ‘280 NAG’). An article coauthored by Felsenfeld and Aldová in 1970 was cited for this strain [11]. In this article, NAG strain ‘280’ is reported as having been ‘isolated in the Mediterranean area.’ Two other strains originating from Aldová were deposited at ATCC by Felsenfeld at the same time: ‘281 NAG’ (catalogue number ATCC 25873) and ‘123 NAG’ (catalogue number ATCC 25874); both these strains are associated with the same article [11] according to the ATCC website. However, other discrepancies between the NCV 10125 and ATCC 25872 strains were noted, including the fact that ATCC 25872 belongs to Heiberg group I (metabolism of sucrose and mannose but not arabinose) rather than group II as originally reported for NCV 10125 [4,10, 11].

Here, we undertook a formal characterization of the strain responsible for the 1965 Czechoslovakian outbreak by sequencing the original NCV 10125 strain deposited in the CNCTC by Aldová in 1966 under catalogue number CNCTC 6536.

## Methods

### Bacterial strain culture

The CNCTC 6536 (= NCV 10125) strain was grown on alkaline nutritive agar and was identified as *V. cholerae* by MALDI-TOF (Bruker, Billerica, MA, USA). Antimicrobial drug susceptibility testing was performed by the disc diffusion (DD) method on Mueller-Hinton agar (Bio-Rad, Marnes-la-Coquette, France) according to the guidelines of the European Committee on Antimicrobial Susceptibility Testing (EUCAST) [12]. The following discs (i2A, Montpellier, France) were used for the DD method: nitrofurantoin (NIT, 100 µg), tetracycline (TET, 30 µg) and meropenem (MEM, 10 µg). The isolates were also tested by the microdilution method (Sensititre, Thermo Fisher Scientific, Cleveland, OH, USA), with ampicillin (AMP), cefotaxime (CTX), azithromycin (AZM), ciprofloxacin (CIP), sulfonamides (SUL), trimethoprim (TMP), trimethoprim-sulfamethoxazole (SXT) and chloramphenicol (CHL). The EUCAST criteria for the interpretation of antimicrobial drug susceptibility testing results for *Vibrio* spp. (v. 14.0) were used when available [12]. For AMP and CHL, the Clinical and Laboratory Standards Institute interpretative criteria for *Vibrio* spp. were used [13]. For TMP and NIT, the EUCAST interpretative criteria for Enterobacterales were used [12]. *Escherichia coli* ATCC 25922 and *Staphylococcus aureus* ATCC 29213 were used for internal quality control.

### Whole genome sequencing

Genomic DNA was extracted with a Genomic-tip 100/G column (QIAGEN, Hilden, Germany) from a 3-h culture grown in tryptic soy broth at 37 °C. It was sent to the Plasmidsaurus sequencing platform (https://www.plasmidsaurus.com/) for sequencing with Illumina (Illumina, San Diego, USA) and Oxford Nanopore Technologies (ONT) (Oxford, UK) techniques.

For Illumina sequencing, the library was prepared with the ExpressPlex Library Prep Kit (seqWell, Beverly, MA, USA) and sequenced on an NextSeq 2000 sequencer (Illumina). Short reads were then filtered with FqCleanER v.23.07 (https://gitlab.pasteur.fr/GIPhy/fqCleanER) to eliminate adaptor sequences and low-quality reads with phred scores <28 and a length <70 bp to generate 70–159 bp paired-end reads.

For ONT sequencing, a PCR-free long-read sequencing library was constructed with the latest v14 library preparation chemistry, including minimal fragmentation of the input genomic DNA in a sequence-independent manner: DNA was selected by 1.0 × ratio paramagnetic bead clean-up without size selection. Sequencing was performed on a PromethION R10.4.1, and the ont-doradod-for-promethion v.7.1.4 base-calling algorithm was used in super-accurate mode. Long reads were then filtered with filtlong v.0.2.0 (https://github.com/rrwick/Filtlong) to remove sequences of less than 5 kb. A *de novo* assembly was generated by Flye v.2.9 [14] to generate two circularised consensus sequences. Medaka v.1.4.4 (https://github.com/nanoporetech/medaka) was used to correct the sequences by a first polishing step with the short reads. A second polishing step with the short reads was performed with Polypolish v.0.5.0 [15]. The two circularized chromosomes generated were annotated with the NCBI Prokaryotic Genome Annotation Pipeline using default parameters (https://www.ncbi.nlm.nih.gov/genome/annotation_prok/) and deposited in GenBank under accession numbers CP147726 and CP147727.

### Genomic sequence analysis

Sequence type (ST) determination was performed with the multilocus sequence type (MLST) scheme of Octavia *et al*. [9]. Serogroup identification was performed with blast v.2.2.26 [16] to compare the *rfb* (O-antigen biosynthesis) gene cluster of CNCTC 6536 with that of the *V. cholerae* O5 reference genome B4202-64 (GenBank accession no. LC594939) and the *V. cholerae* O37 reference genome 1322–69 (GenBank accession no. LC594925) [17]. Other genetic markers were analysed with blast v.2.2.26 against whole-locus reference sequences for *Vibrio* pathogenicity islands (VPI-1 and VPI-2), *tcpA*, the major pilin subunit of the toxin-coregulated pilus (TCP) and the *ctxA* and *ctxB* genes encoding the cholera toxin (CT)*,* as previously described [18]. The presence and type of acquired antibiotic resistance genes (ARGs) or ARG-containing structures were determined with ResFinder v.4.0.1 [19] and PlasmidFinder v.2.1.1 [20]. The presence of genetic mutations resulting in resistance to quinolones (*gyrA, parC*) and resistance to nitrofurans (*nfsA*, nitroreductase, VC0715 and VCA0637, dihydropteridine reductase) was investigated by manual analysis of the sequences assembled *de novo* with blast, as previously described [18,21].

### Additional genomic data

Raw sequence files and assembled genomes from 387 *Vibrio* strains were downloaded from the European Nucleotide Archive (https://www.ebi.ac.uk/ena/) and GenBank (https://www.ncbi.nlm.nih.gov/genbank/). These genomes comprised the 383 genomes described by Dorman *et al*. [22] and genomes from *V. cholerae* O5 reference strain B4202-64, *V. cholerae* O37 reference strain 1322–69 and the ATCC 25872 and ATCC 25874 strains [17,23]. No genomic sequence was found for the ATCC 25873 strain in the various genome databases. All 388 genomes (including CNCTC 6536) used in this study are listed, with their accession numbers, in Table S1, available in the online version of this article.

### Phylogenetic analyses

The paired-end reads and draft or assembled genomes were mapped onto the *V. cholerae* O1 N16961 reference genome (GenBank accession numbers AE003852 and AE003853) with snippy v.4.6.0 (https://github.com/tseemann/snippy). The resulting single-nucleotide variant (SNV) alignment was used to generate the final phylogenetic tree after 200 bootstraps with IQ-TREE v.2.2.2.2 under the GTR model [24]. This global tree was rooted in the genomes of *V. metoecus* (07–2435 and RC341) and *V. parilis* (RC586) (Table S1) and was visualized with iTOL v.6 [25].

## Results and discussion

After circularization, *V. cholerae* strain CNCTC 6536 (= NCV 10125) contained two chromosomes, the larger one (GenBank accession no. CP147726) being 2 954 495 bp in size and the smaller one (GenBank accession no. CP147727) being 1 123 164 bp in size. This strain lacked classical virulence factors, such as the CT *ctxA* and *ctxB* genes; the TCP *tcpA* gene; and the pathogenicity islands, VPI-1 and VPI-2. No antibiotic resistance genes were detected in its genome, consistent with the lack of phenotypic antibiotic resistance observed for the CNCTC 6536 strain.

**Fig. 1.**
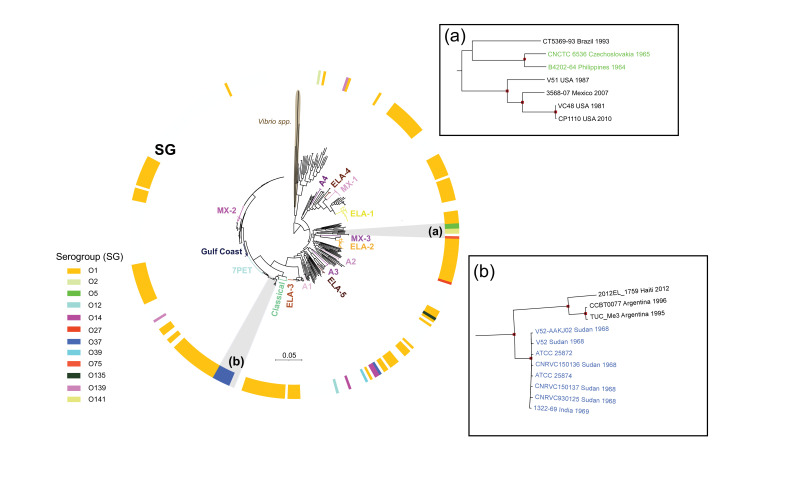
Maximum likelihood phylogeny of 388 *Vibrio* genomes, including *V. cholerae* CNCTC 6536 (= NCV 10125). Three *Vibrio* genomes belonging to non-*V. cholerae* species (07–2435, RC341 and RC586) were used as the outgroup (brown). The colour coding of the ring indicates the serogroup of the isolates. Lineages are named according to previous reports [22,33]. A scale bar indicates the number of nucleotide substitutions per variable site. A magnification of clades containing serogroups O5 and O37 (a and b, respectively) is shown on the right. In panels a and b, the name, country and year of isolation are indicated for each genome at the tips (in green for serogroup O5 strains and in blue for O37) and the bootstrap values ≥90 % are shown in red.

It was inferred that the CNCTC 6536 strain belonged to serogroup O5 [100% query coverage, 99% identity (22 915/22 918) and no gap in the alignment with the *rfb*O5 reference sequence], whereas query coverage was only 8% [97.4 % identity (2356/2453) with the *rfb*O37 reference sequence (29 519 nucleotides)]. This strain belonged to multilocus sequence type ST8, the same ST as the *V. cholerae* O5 reference strain (B4202-64) of the Sakazaki serogroup type strains [1,2]. The CNCTC 6536 and B4202-64 strains clustered together (pairwise SNV difference of 3215 SNVs) on the *V. cholerae* phylogenetic tree based on 92 174 SNVs (Fig. 1). The serogroup O5 reference strain (B4202-64) was isolated from patients with diarrhoea and their contacts during field studies in the Philippines in 1964 [26,27]. This serogroup accounted for 8.9% (18/203) and 9.9% (20/202) of NAG *V. cholerae* strains isolated in the Philippines and India, respectively, before 1970 [27].

By contrast, *V. cholerae* O37 strains ATCC 25872 and ATCC 25874 both carried *ctx* genes, the *tcpA* gene, VPI-1 and VPI-2 [28]. The ATCC 25872 and ATCC 25874 strains are reportedly not identical according to their rRNA intergenic spacer region sequences [29], but they both belong to ST68 and to the same cluster as the *V. cholerae* O37 strains responsible for the 1968 Sudanese outbreak [30], which included strains CNRVC930125 (original name ‘13030’), CNRVC150136 (13026) and CNRVC150137 (13031/1) (Fig. 1). All three of these strains were sent to the Institut Pasteur in 1969 for inclusion in the institute’s collection. The *V. cholerae* O5 and O37 strains were distant on the *V. cholerae* phylogenetic tree, with serogroup O37 strains being closely related to the toxigenic *V. cholerae* O1 strains of the classical biotype and O5 strains related to toxigenic *V. cholerae* O141 strains (Fig. 1) [31,33].

So how could this confusion have arisen? These three ATCC strains (25872, 25873 and 25874) all originated from the laboratory of Aldova (who had described the Czechoslovakian outbreak). It is therefore possible that the origin of these strains was misattributed to the 1965 outbreak in Czechoslovakia. The mention of a geographic origin in ‘the Mediterranean area’ in the associated metadata on the ATCC website is rather vague but not consistent with a Czechoslovakian origin. A search of the ATCC archives revealed that these three strains, deposited by Felsenfeld on 6 July 1970, were obtained from patients with symptoms of cholera in an Arab country in the Near or Middle East in 1968 or 1969. It was indicated that this country had not reported the outbreak and did not wish to be named. A search of the CNCTC archives revealed that three NAG strains (original names 13026, 13 030 and 13031/1; the same strains received by the Institut Pasteur in 1969) from a cholera-like outbreak in Sudan in 1968 were received by Aldová on 3 December 1968 (from Dr D. Barua, WHO Geneva). The third Sudanese strain (13031/1) had been labelled NAG ‘282’ (actually a lyophilization name given on 28 December 1968) at the CNCTC. Due to missing data, it was not possible to find the lyophilization names of the two first Sudanese strains. However, we strongly suspect they were labelled NAG ‘280’ and ‘281,’ the original names of ATCC 25872 and ATCC 25873, respectively. The terminology used by Felsenfeld *et al*.–‘from patients in the Mediterranean area’ [11] – can be explained by the request from the Ministry of Health of Sudan in 1968 for the World Health Organization (WHO) assistance to investigate this outbreak. This assistance was initially provided by the WHO Regional Office for the Eastern Mediterranean (EMRO), which includes Sudan [30]. Therefore, all these findings are consistent with *V. cholerae* O37 strains 280 (ATCC 25872), 281 (ATCC 25873) and 282 being isolated during the outbreak of severe gastroenteritis that occurred in the El Gedaref district of Sudan in November 1968 following the contamination of a recently opened Artesian well that attracted tens of thousands of people due to its reputed ability to cure various diseases [30].

Another discrepancy is that the third ATCC strain – ATCC 25874, linked to the same geographic area (probably Sudan) as the other two – was not originally numbered ‘282’ but ‘123’. We found in the CNCTC archives that the 1965 Czechoslovakian outbreak strain NCV 10125 was given a lyophilization name of ‘123’ (like the original name of ATCC 25874) at the CNCTC on 14 January 1966. However, in our phylogenetic analysis, ATCC 25874 was not grouped with the original *V. cholerae* O5 strain NCV 10125 from Czechoslovakia but with the *V. cholerae* O37 strains isolated (without a doubt) during the 1968 Sudanese outbreak. As ATCC 25874 grouped with the *V. cholerae* O37 strains, this suggests that the original name of the strain provided by Felsenfeld during the deposition of the ATCC 25874 strain was erroneous, the correct name being ‘NAG 282’.

In conclusion, by sequencing the original representative strain of the 1965 Czechoslovakian outbreak [CNCTC 6536 (= NCV 10125)], we show that, contrary to previous claims, the strain responsible for this outbreak was a *V. cholerae* O5 strain that did not carry the principal virulence factors of *V. cholerae*.

## supplementary material

10.1099/mgen.0.001282 Uncited Table S1.

## Abbreviations

ARGs: antibiotic resistance genes
ATCC: American Type Culture Collection
CNCTC: Czech National Collection of Type Cultures
CT: cholera toxin
DD: disc diffusion
EUCAST: European Committee on Antimicrobial Susceptibility Testing
NAG: non-agglutinable
NCV: non-cholera *Vibrio*
ONT: Oxford Nanopore Technologies
SNV: single-nucleotide variant
ST: sequence type
TCP: toxin-coregulated pilus
VPI: *Vibrio* pathogenicity islands
WHO: World Health Organization
